# Video-electroencephalographic findings and clinical characteristics of bathing seizures in children

**DOI:** 10.3389/fneur.2024.1366206

**Published:** 2024-02-19

**Authors:** Xiaojun Kuang, Hongmei Liao, Hongjun Fang, Xiao Zhang, Lijuan Wang, Liming Yang, Liwen Wu

**Affiliations:** Hunan Children's Hospital, Changsha, Hunan, China

**Keywords:** bathing epilepsy, reflective epilepsy, video EEG, children, SMC1A

## Abstract

**Objective:**

To explore the electroencephalogram (EEG) and clinical characteristics of childhood bathing epilepsy.

**Methods:**

We conducted a prospective summary of the clinical data from 10 children with bathing epilepsy who were admitted to Hunan Children’s Hospital from April 2019 to November 2023 and analyzed their EEGs and clinical characteristics.

**Results:**

Our 10 patients included eight males and two females, with seizure-onset ages ranging from 4 months and 20 days to 14 months. Nine cases showed normal intellectual development, and one case manifested delayed development. The Video-EEG (VEEG) findings showed that nine cases exhibited normal background with no interictal epileptic discharge. The seizures were characterized by lip cyanosis, tachycardia or bradycardia, weakness, paleness, and loss of consciousness. Ictal EEG revealed rhythmic fast waves, spike waves, spike-slow waves, or slow and sharp-wave activity over the temporal region (eight cases) or the occipital and temporal regions (one case), finally evolving into a delta rhythm that lasted for 57–201 s. These children exhibited no seizures after discontinuing bathing and were not administered antiseizure medication. The interictal EEG of one case reflected mild slow background and focal interictal epileptic discharge; and her semiology was eyes gazing to right, with clonic movements of the right face and lips, lip cyanosis, bradycardia, and impaired consciousness. Ictal EEG showed spike–wave and spike-slow-wave rhythms over the left central, parietal, and temporal regions; these then spread to the left hemisphere, lasting for approximately 104 s. This patient did not exhibit bathing seizures after stopping her bathing but later experienced frequent spontaneous and drug-resistant seizures. The interictal EEG background slowed down, while focal epileptic discharge increased. Her intellectual development was significantly delayed, and a novel pathogenic mutation in the SMC1A gene, c.298+2T>C, was detected. She was diagnosed with developmental and epileptic encephalopathy.

**Conclusion:**

A majority of children with bathing epilepsy in our study showed focal autonomic seizures accompanied by impaired consciousness. Stopping bathing could control the seizures and showed a good prognosis. A few infants manifested a poor prognosis, and we posit that bathing seizure rarely constitute the early manifestations of developmental and epileptic encephalopathy. VEEG findings and clinical features can also indicate the prognosis.

## 1 Introduction

Bathing epilepsy refers to a specific type of reflex epilepsy caused by bathing susceptible children in water that is nearly body temperature (36°C–37°C). Both bathing seizures and hot-water seizures constitute reflex epilepsy, and some patients experience spontaneous seizures (1). However, bathing epilepsy is different from hot-water epilepsy, as in most hot-water epilepsy cases, reducing the water temperature can prevent seizures; and most cases reflect genetic factors (2, 3). However, in bathing epilepsy—with a fixed water temperature—there may be other triggering factors such as the pressure of water on the buttocks, abdomen, face, or other parts of the body. Bathing epilepsy is more common in infancy, and most infants show a good prognosis and do not require antiseizure medication (1). Although a majority of cases of bathing epilepsy are benign, most exhibit autonomic nervous system symptoms that can affect the cardiovascular system and pose a risk of sudden unexpected death in epilepsy (SUDEP) (4). Early diagnosis is therefore of paramount importance. There are currently relatively few reports on the clinical characteristics, seizure semiology, treatment, or prognosis of bathing epilepsy, particularly with respect to reports of interictal electroencephalography (EEG) (5, 6). In this article, we recorded the EEG characteristics of 10 cases of seizures that occurred during warm-water bathing and summarized their clinical characteristics and follow-up. We posit that this study will provide a reference for physicians that will allow for early diagnosis, correct prognostic judgment, and appropriate treatment.

## 2 Clinical data and methods

Personnel at our EEG monitoring center in our Neurology Department bathed those children who only experienced seizures while taking a bath during the VEEG monitoring process with the consent of the children’s guardians. We enrolled a total of 10 pediatric patients who only experienced reflex epilepsy while taking a bath from April 2019 to November 2023; seizures were detected in all of the infants while in a warm-water bath during EEG monitoring, and these 10 children were then diagnosed with bathing epilepsy. We excluded infants with some non-epileptic conditions such as syncope, breath-holding spells, alternating hemiplegia of childhood (AHC), hyperekplexia, and paroxysmal extreme pain disorder (PEPD).

Bathing was implemented and recorded as follows. We used a 32-channel VEEG monitor (Nihon Kohden EEG-1200C), and 19 channel recording electrodes were placed according to the international 10–20 system. All EEG connections criteria were met. We conducted electrocardiographic (EKG) and bilateral deltoid muscle electromyographic recordings. We performed passive eye-opening and closing tests and intermittent flash stimulation tests during the conscious phase in all 10 patients. We adjusted the room temperature to 25°C–28°C and added warm water at 36°C–37°C to approximately half of the basin (i.e., so that the water level was at the baby’s navel), removed the child’s clothing, placed the child in the basin, marked the time the child entered the water, and observed and recorded the semiology and EEG findings of the child during the seizure. We continued monitoring after the seizure, recording at least one sleep cycle until 30 min after self-awakening, with a monitoring time of 4–15 h. We separate water and electricity during the bathing process for the sake of electrical safety.

We collected information as to the children’s age at onset, sex, current medical history, past history, family history, a physical examination, head magnetic resonance imaging (MRI), cardiac ultrasonography, routine blood chemistry and biochemistry, blood tandem mass spectrometry and urine gas chromatography–mass spectrometry, Gesell developmental scale, and other data from the 10 pediatric patients and summarized and analyzed them.

## 3 Results

### 3.1 Demographics and clinical presentation of bathing epilepsy patients

Of the 10 patients, eight were males (80%) and two were females (20%). All patients developed seizures that occurred only during routine bathing, with the onset age ranging from 4 months and 20 days to 1 year and 2 months. One child had a fever for 7 days before the seizure, and two children had diarrhea or pneumonia during the seizure. The remaining children exhibited no obvious cause prior to the seizures. Two of the 10 patients had a family history of convulsions. The seizure symptoms of nine children were similar, with symptoms such as lip cyanosis, tachycardia or bradycardia, weakness, paleness, and loss of consciousness appearing 3.5–10 s after the start of bathing. Two children also exhibited oral automatistic movements. One case (patient 10 in Table 1) showed eyes gazing to right, clonic movements of the right face and lips, lip cyanosis, bradycardia, and impaired consciousness 5 s after taking a shower. Three children manifested spontaneous seizures, and one child (patient 4 in Table 1) had rhythmic clonic seizures in the right upper limb that lasted for several seconds before bathing seizures but which resolved spontaneously; there was no change in facial color or impaired consciousness, with 0–5 episodic seizures per day. The attack then completely stopped before the bathing seizures. One case (patient 5 in Table 1) had one unprovoked episode of lip cyanosis and limb stiffness during sleep after bathing seizures, lasting for about 10 s. One case (patient 10 in Table 1) revealed frequent and gradual spontaneous seizures after 15 days of bathing-seizure onset. The 10 pediatric patients exhibited normal cardiac ultrasonographic scans, typical genetic and metabolic screening results of blood and urine, and routine blood electrolytes. Brain imaging was abnormal in three cases. The bilateral frontal and temporal extra brain spaces were slightly wider and the bilateral ventricles were fuller in case 5. Case 7 showed slightly wider bilateral frontal and temporal extra brain spaces, slightly enlarged and deformed bilateral ventricles, and slightly thinner corpus callosum. Case 10 demonstrated an arachnoid cyst and slightly widened bilateral frontal and temporal extra-brain spaces. Follow-up was from 2 months to 4 years and 8 months. When patients 1–9 stopped taking baths in the bathtub and a change was made in their style of bathing, there were no seizures. After about 1 week to 1 month, the infants gradually transitioned to warm-water baths without similar episodes. These nine patients did not take any antiseizure medications (detailed clinical data of the 10 patients are shown in Table 1).

**Table 1:** Clinical characteristics and follow-up of children with bathing seizures.

Pt	Sex	Age at onset	Early symptoms before bathing seizures	Seizure frequency	Brain MRI	Score of Gesell scale	Spontaneous seizure	Family history	ASMs	Age at last follow-up	Response to therapy
1	Male	1 year 2 months	No	7 episodes/15 days	Normal	92	No	Grandmother	No	4 years 9 months	Seizure-free
2	Female	10 months	Fever	4 episodes/5 days	Normal	94	No	No	No	4 years 7 months	Seizure-free
3	Male	10 months	No	4 episodes/10 days	Normal	95	No	No	No	4 years 7 months	Seizure-free
4	Male	8 months 29 days	No	4 episodes/9 days	Normal	95	Yes	No	No	3 years 6 months	Seizure-free
5	Male	5 months	No	8 episodes/4 months	Bilateral frontal and temporal extra-brain spaces were slightly wider, and bilateral ventricles were fuller	90	Yes	No	No	3 years 8 months	Seizure-free
6	Male	8 months	No	4 episodes/20 days	Normal	93	No	No	No	2 years 8 months	Seizure-free
7	Male	8 months	No	3 episodes/10 days	Bilateral frontal and temporal extra-brain spaces were slightly wider, bilateral ventricles were slightly enlarged and deformed, and the corpus callosum was slightly thinner	86	No	Father	No	2 years 6 months	Seizure-free
8	Male	9 months 3 days	Diarrhea	3 episodes/4 days	Normal	92	No	No	No	4 months	Seizure-free
9	Male	6 months 28 days	No	4 episodes/20 days	Normal	93	No	No	No	2 months	Seizure-free
10	Female	4 months 20 days	Diarrhea	4 episodes/5 days	Bilateral frontal and temporal extra-brain spaces were slightly widened	70	Yes	No	LEV VPA TPM	2 years	Frequent seizures
											Moderate developmental delay
											Developmental epileptic encephalopathy

### 3.2 VEEG findings

**Figure 1:**
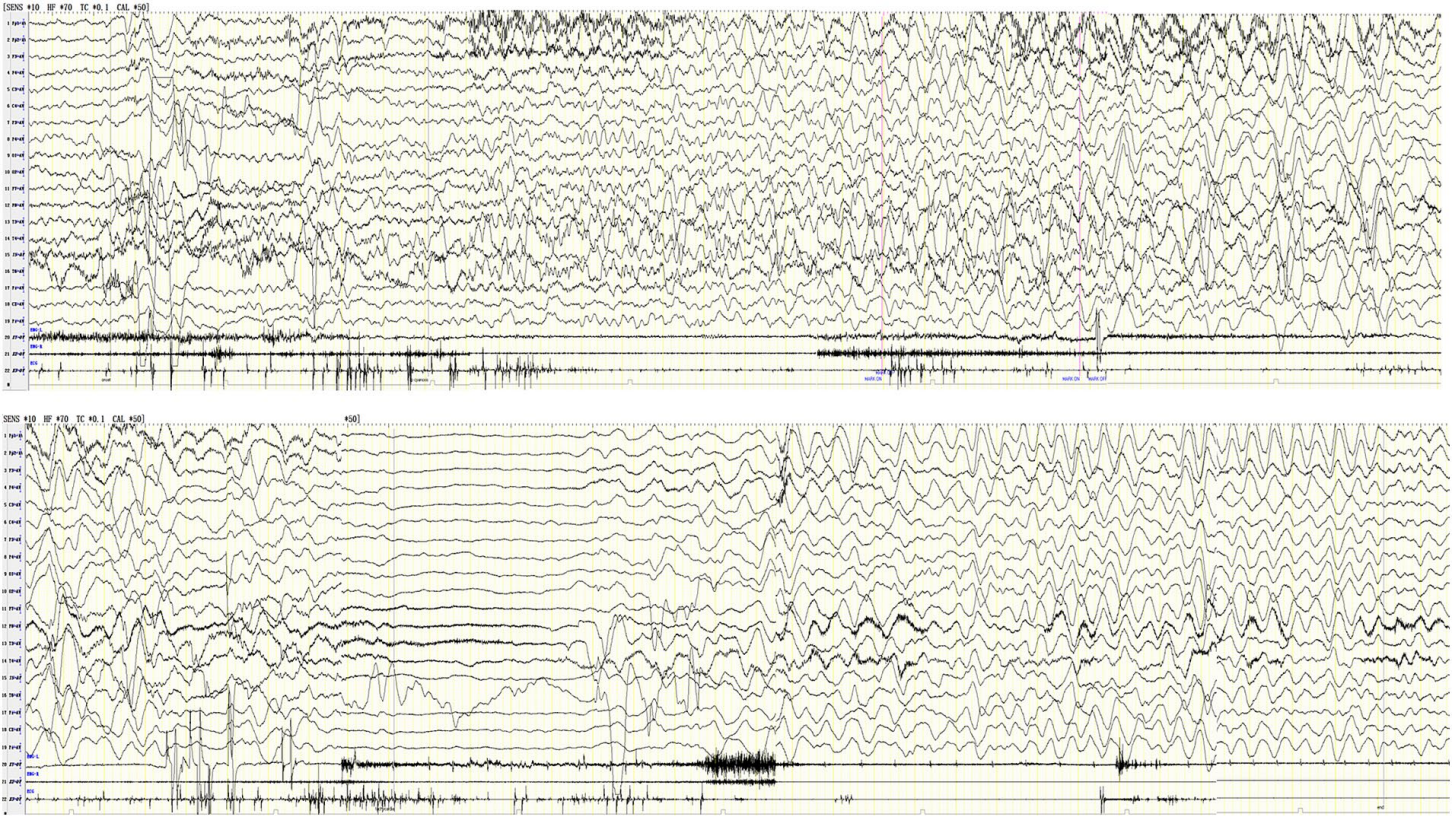
Ictal EEG of case 8 shows left temporal region low-medium amplitude rhythmic sharp-slow wave → diffuse high-amplitude delta rhythm in various regions → generalized suppression → diffuse high-amplitude delta rhythm in various regions, lasting for 201 s (X1 left deltoid muscle, X2 right deltoid muscle, X3-EKG drop).

**Figure 2:**
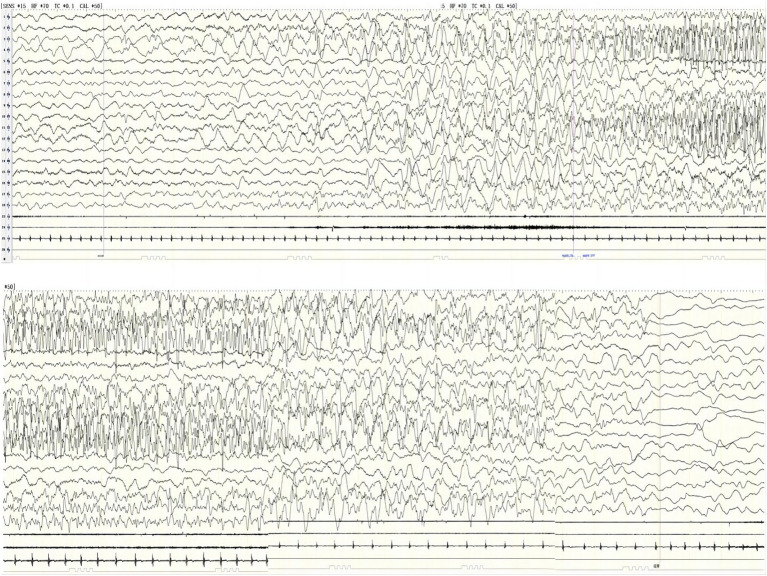
Ictal EEG of case 10 shows left central, parietal, and temporal spike-slow wave rhythm → high-amplitude slow waves in each region → high-extremely high amplitude spikes and spike-slow wave in the left hemisphere, spread midline region, lasting for 104 s (X1-left deltoid muscle, X2-right deltoid muscle, X3-EKG).

Nine cases showed a normal background rhythm, while one patient showed a slight slowing of waves (case 10). Interictal EEG demonstrated small spikes in the bilateral Rolandic area including central, parietal, and posterior temporal regions during sleep in one patient and a small number of sharp waves in the left frontal, central, parietal, and temporal regions in another patient. Ictal EEG recordings revealed rhythmic changes beginning in the left temporal region in five patients, the right temporal region in three patients, and the right occipitotemporal region in one patient. The initial discharge modes of ictal EEG included low-amplitude fast rhythm (four cases); low-amplitude spike and mixed medium-amplitude slow waves or spike slow-wave rhythm (two cases); and low to medium amplitude theta waves and a sharp wave rhythm (three cases); the latter gradually evolved into diffuse high to extremely high-wave amplitude delta rhythms in various regions. Ictal EEG recordings of patient 8 showed low to medium amplitude spike slow-wave rhythm over the right temporal area that evolved into a diffuse high-amplitude delta rhythm, followed by widespread voltage suppression, and finally a diffuse high-amplitude delta rhythm (see Figure 1). Ictal EEG lasted for 57–201 s. The overall characteristic of the ictal EEG was low amplitude, and it did not exhibit any prominent spikes, while the high-amplitude delta rhythm was prominent and monomorphic. The ictal EEG of patient 10 showed left central, parietal, and temporal spike slow-wave rhythm in the first 20 s, gradually evolving into high-amplitude slow waves significant in each region; then high to extremely high amplitude spikes and spike slow-wave rhythm in the left hemisphere; and finally extending to the midline region, lasting for 104 s (see Figure 2; the details of VEEG in our 10 pediatric patients are shown in Table 2).

**Table 2:** VEEG findings of bathing seizures in children.

Pt	Age	Background	Interictal epileptic discharge	Latency after initiation of bathing	Semiology	Ictal EEG	Duration of seizure in VEEG	Background rhythm after seizure
1	1 y 2 m	Normal	No	4 s	Motor arrest, lip cyanosis, tachycardia (105/min → 160/min), pallor, impaired consciousness	Left temporal region low-amplitude spike mixture medium amplitude delta wave → diffuse medium-high-medium amplitude delta rhythm in various regions	157 s	Generalized slow waves
2	10 m	Normal	No	7 s	Motor arrest, bradycardia (120/min → 90/min), lip cyanosis, impaired consciousness	Right temporal region medium-amplitude rhythmic theta and sharp wave → diffuse medium-high amplitude delta rhythm in various regions	68 s	Generalized slow waves
3	10 m	Normal	No	10 s	Motor arrest, lip cyanosis, tachycardia (120/min → 180/min), pallor, impaired consciousness	Left temporal region medium-amplitude rhythmic theta and sharp wave → diffuse medium-high amplitude delta rhythm in various regions	70 s	Generalized slow waves
4	9 m	Normal	No	10 s	Lip cyanosis, tachycardia (120/min → 180/min), oral automatisms, limpness, pallor, impaired consciousness	Left temporal region low-amplitude rhythmic fast wave → diffuse extremely high amplitude delta rhythm in various regions	95 s	Generalized slow waves
5	9 m	Normal	Small spike in Rolandic region	6 s	Lip cyanosis, bradycardia (120/min → 90/min), limpness, impaired consciousness	Left temporal region low-amplitude rhythmic fast wave → diffuse high-extremely high amplitude delta rhythm in various regions	115 s	Generalized slow waves
6	8 m	Normal	No	5–6 s	Lip cyanosis, tachycardia (115/min → 180/min), pallor, oral automatisms, limpness, impaired consciousness	Right temporal region low-amplitude rhythmic fast wave → diffuse extremely high amplitude delta rhythm in various regions	168 s	Generalized slow waves
7	8 m	Normal	No	6 s	Lip cyanosis, limpness, Bradycardia (120 → 90/min) impaired consciousness	Right temporo-occipital region medium-amplitude rhythmic theta and sharp wave → diffuse medium-high amplitude delta rhythm on the right side	57 s	Generalized slow waves
8	9 m 7 d	Normal	No	3.5 s	Lip cyanosis, Bradycardia (125/min → 80/min), wandering gaze, staring gaze, limpness, pallor, impaired consciousness	Left temporal region low-medium amplitude rhythmic sharp-slow wave → diffuse high-amplitude delta rhythm in various regions → generalized suppression → diffuse high-amplitude delta rhythm in various regions	201 s	Generalized slow waves
9	7 m 19 d	Normal	No	9 s	Lip cyanosis, Bradycardia (120/min → 90/min), limpness, impaired consciousness	Left temporal region low-amplitude rhythmic fast wave → diffuse medium-high amplitude delta rhythm in various regions	92 s	Generalized slow waves
10	4 m 22 d	Slightly slow	Sharp in the frontal, central, parietal, and temporal regions	5 s	Eyes gazing to the right, clonic movements of the right face and lips, lip cyanosis, Bradycardia (140/min → 85/min), impaired consciousness	Left central, parietal, and temporal spike-slow wave rhythm → high-amplitude slow waves in each region → high-extremely high amplitude spikes and spike-slow wave in the left hemisphere, spread midline region	104 s	Rhythmic delta slow waves over the left hemisphere

**Figure 3:**
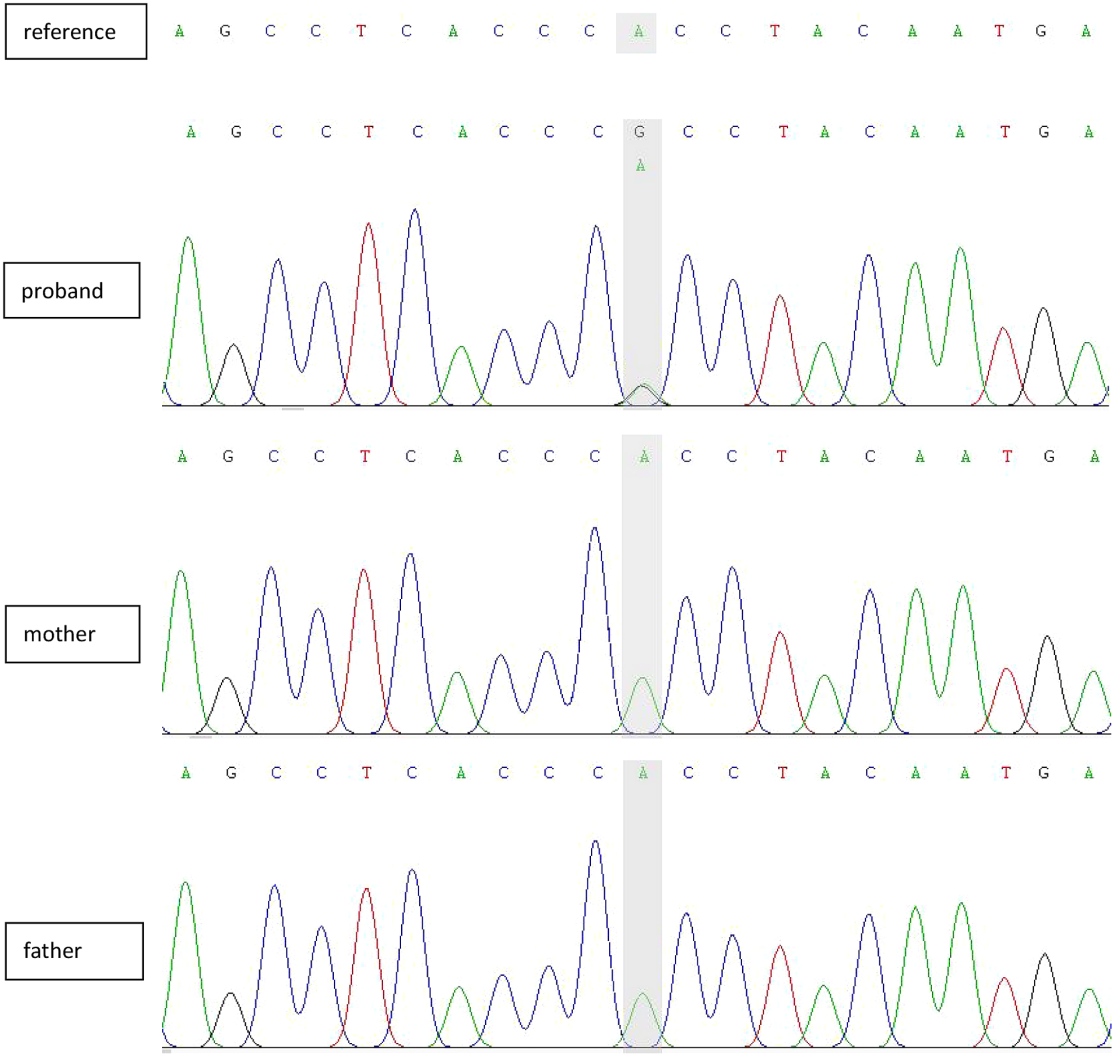
Sanger-sequencing electropherograms showing a *de novo* splicing variant in SMC1A (NM_006306.4:exon2:C.298+2T>C).

Patient 10 showed mild developmental delay, with no deformities of the head or face; her left middle finger, ring finger, and little finger were flexed; and her brain MRI was abnormal. The seizure form comprised obvious focal motor symptoms. The VEEG showed a slightly slow-wave background and a small amount of focal interictal epileptiform discharge. The ictal EEG was typical of focal and regional spikes, with spike slow waves that persisted; and after the seizure, the EEG showed a unilateral delta rhythm. Considering that the initial four seizures were induced by bathing, antiseizure medications were not prescribed. The patient subsequently experienced spontaneous clustered seizures, occurring as 10 episodes per day and they were treated with levetiracetam. She then developed recurrent episodes of clonic movements of the right face, lips, and limb at a rate of approximately one episode per minute. After continuous administration of midazolam, the seizures stopped, but there were still recurrent episodes after halting midazolam. Multiple EEGs indicated a slow background rhythm and multifocal sharp waves. Using whole-genome sequencing, we detected a c.298+2T>C mutation in the SMC1A gene (Figure 3). Topiramate and valproate were added, and the patient was followed-up with for 2 years, during which the patient still exhibited spontaneous seizures. The intellectual development of this child was moderately delayed, and the diagnosis was developmental epileptic encephalopathy.

## 4 Discussion

Reflex epilepsy refers to recurrent seizures primarily caused by specific motor, sensory, or cognitive stimuli, including both simple sensory stimuli and complex stimuli (7–10). In this article, we reported 10 cases of bathing epilepsy in which the bath water was nearly body temperature, thus belonging to the category of simple sensory stimulation. The age at onset for our infants was from 4 months and 20 days to 14 months, with a latent period of 3.5–10 s. Of these patients, boys accounted for 80%, which was consistent with previous reports.

Some literature suggests that bathing epilepsy is different from hot-water epilepsy (1). Hot-water epilepsy is related to the temperature of the water (ranging from 38°C to 55°C) during bathing, and lowering the water temperature during bathing reduces seizure incidence or no longer induces epilepsy at all (11). Most reports of hot-water epilepsy are from India and Turkey where ritual bathing involves repeatedly pouring hot water over the child’s head, suggesting a genetic predisposition. A genetically aberrant thermoregulatory system or an anatomical abnormality of the temporo-insular and parietal networks has been suggested as a possible mechanism for epileptogenesis. Hot-water epilepsy are usually self-limiting, and even if antiseizure medications are required, seizure-free status can be achieved with monotherapy (12). Thermal sensitivity is an important characteristic of children with Dravet syndrome. Patients with Dravet syndrome may exhibit hot bathing epilepsy in the early stages (13). Lee et al. (14) reported 26 patients with Dravet syndrome exhibiting hot water bath test induced seizures, the median body temperature was 37.8°C, ranging from 36.8°C to 38.7°C, the median water temperature was 39.0°C, ranging from 37.9°C to 40.0°C. Hot bathing epilepsy in patients with Dravet syndrome is related to water temperature, and slight rise in body temperature around 37°C–37.9°C may induced seizure. These patients usually have a poor prognosis and later develop into refractory epilepsy, cognitive impairment, and motor disorders. While bathing epilepsy is unrelated to water temperature. In our study, the temperature of the water was lukewarm (36°C–37°C) and there was no clear relationship between seizures and pouring water over the infant’s head, thus excluding hot-water epilepsy. In our practice, we have found that seizures are not triggered when a baby’s face and limbs come into contact with warm water, but it is only when the buttocks and abdomen are immersed in water that seizures are triggered. Wiping the infant’s body rather than bathing the infant in warm water does not induce seizures. Therefore, seizures may be related to the pressure stimulation of water on the buttocks and abdomen.

There are few extant studies on EEGs of bathing seizures, and most do not entail ictal recordings—possibly due to the fact that VEEG recordings are difficult to obtain during a bath. For example, Dashi et al. (15) reported a 10-year-old boy with normal psychomotor development. His interictal EEG demonstrated epileptiform discharges in the right frontal-anterior temporal region consisting of spike–wave complexes in brief runs of 1–3 Hz. Changing the style of bathing then resulted in freedom from seizures at the two-year follow up. Gorostidi et al. (1) reported on two cases and also reviewed the literature for ictal VEEG of 16 children with respect to bathing seizures. Ictal VEEG revealed delta-theta high-amplitude focal waves that involved temporal and adjacent regions, with a rapid spread to the ipsilateral hemisphere or generalization. In our analysis, we detected one episode of bathing seizure during VEEG in 10 pediatric patients. Seizures in nine cases were of focal onset with loss of awareness and prominent autonomic symptoms such as lip cyanosis, slowed or increased heart rate, pallor, and no secondary generalized tonic–clonic seizure. Ictal VEEG revealed fast rhythms (four cases), spike slow-wave or spike mixed slow-wave rhythms (two cases), and theta or sharp-wave rhythms (three cases) involving the temporal region (five cases on the left and three cases on the right) and the right occipital and temporal areas (one case); these gradually evolved into diffuse delta rhythms in various regions. According to the ILAE2017 Epilepsy Classification (16), these children were diagnosed with focal autonomic epilepsy with impaired awareness that may have been due to the stimulation of peripheral receptors by water temperature and pressure during bathing and the activation of the autonomic nervous system network in the medial temporal lobe and insula through afferent nerves (17, 18). All of these nine children became seizure free by changing their style of bathing at two-month-to-four-year and eight-month follow-ups, and psychomotor development was noted to be normal. Patient 10 developed episodes of a staring gaze, clonic movements of the right face and lips, cyanosis, bradycardia, and impaired consciousness. Ictal EEG showed a spike slow-wave rhythm starting from the left central, parietal, and temporal regions and gradually evolving into a spike slow-wave rhythm in the left hemisphere that ultimately affected the midline area. After termination of bathing, the child remained episode-free for a period of time. Unfortunately, frequent and uncontrollable spontaneous seizures appeared after 15 days, with delayed development, and the child was diagnosed with developmental epileptic encephalopathy.

In our investigation, the youngest onset age for patients 1–9 was 4 months and 20 days, and the oldest onset age was 1 year and 2 months. Gorostidi et al. (1) described 16 infants with bathing seizures with an onset age of 4–24 months (excluding one case of onset at 36 months and one case of neonatal onset). Thus, bathing epilepsy was more common in infants under 2 years of age and exhibited significant age-dependence. Our patient exhibited seizures 3–8 times from 4 days to 4 months, with seizures mainly concentrated within a certain period of time and not occurring at other times, and they exhibited distinctly clustered seizure characteristics. The age-dependent and clustered characteristics of bathing seizures may be related to the excessive excitation of physiological stimuli in cortical or subcortical neuronal regions, and the specific underlying mechanism needs to be studied further (4). The types of seizures in these infants were focal autonomic seizures with impaired consciousness, and the interictal EEGs were basically normal (one case showed spike waves involving the Rolandic region). The ictal EEG is characterized by various rhythmic electrical activities in the temporal area, which gradually evolve into diffuse, non-persistent high-amplitude delta rhythms. Neuroimaging was basically normal (two cases showed mild abnormalities), with normal neural development. Two infants in this study possessed a family history of seizures. We posit that avoiding triggering factors will control seizures and Gorostidi et al. (1) reported carbamazepine, sodium valproate, and levetiracetam are also beneficial, portending a favorable long-term prognosis. The clinical and EEG characteristics of patients 1–9 were generally consistent with benign infantile epilepsy (familial or non-familial), which was defined as self-limited familial and non-familial infantile epilepsy (SeLIE) in the 2022 ILAE Classification for Epilepsy Syndromes. The difference here was that these children had bathing-induced epilepsy, but we ask, “Did they reflect a special type of SeLIE (19)?”

Previous studies have shown genetic changes in a small number of patients with bathing epilepsy. Although genetic factors contribute to the etiology of bathing epilepsy, the molecular mechanism (s) underlying this disease remains arcane. Braun et al. (20) diagnosed bathing epilepsy in an MDB5-associated neurological disorder (MAND) patient with a chromosome 2q22.3q23.2 deletion. Accogli et al. (4) identified eight distinct pathogenic or likely pathogenic variants and two variants of unknown significance in SYN1 in eight families with bathing epilepsy. Unfortunately, only patient 10 in our study underwent genetic testing due to recurrent seizures and poor treatment outcomes. In our study, symptoms in nine patients were benign and self-limited. Patient 10 was characterized as experiencing bathing epilepsy, followed by spontaneous, untriggered seizures that ultimately resulted in drug refractoriness and progressive worsening of the syndrome. Whole-genome sequencing identified a c.298+2T>C mutation in the SMC1A gene. The SMC1A gene mutation was first described in a patient with rare Cornelia de Lange syndrome (CdLS), characterized by growth retardation and typical facial deformities (21). There have also been recent reports of phenotypes such as seizures, developmental epileptic encephalopathy, or Rett syndrome (22, 23). The phenotypic characteristics of the patient in this particular case were developmental delay, finger deformity, a slow EEG background, multifocal interictal discharges, and uncontrollable focal seizures—all of which were consistent with developmental epileptic encephalopathy. We know of no previous reports of SMC1A gene variant phenotypes in infants in whom bathing epilepsy characterized their first epileptic episode.

## 5 Conclusion

In summary, a majority of cases of infantile warm-water bathing epilepsy are self-limited and exhibit a good long-term prognosis. A few infants manifest the initial symptoms of developmental epileptic encephalopathy, with developmental delay occurring before onset, with abnormal head imaging; obvious symptoms of focal motor seizures; persistent, drug-resistant, or clustered spontaneous seizures; abnormal VEEG background; interictal discharge; and typical and persistent focal spikes and slow waves in ictal EEG. These characteristics often suggest a potentially poor prognosis, and we recommend that antiseizure medications be administered early and that genetic testing be conducted.

## Data availability statement

The datasets presented in this article are not readily available because of ethical and privacy restrictions. Requests to access the datasets should be directed to the corresponding authors.

## Ethics statement

The studies involving humans were approved by the Ethics Committee of Hunan Children’s Hospital. The studies were conducted in accordance with the local legislation and institutional requirements. Written informed consent for participation in this study was provided by the participants' legal guardians/next of kin. Written informed consent was obtained from the individual(s), and minor(s)' legal guardian/next of kin, for the publication of any potentially identifiable images or data included in this article.

## Author contributions

XK: Data curation, Writing – review & editing, Formal analysis, Project administration, Validation, Writing – original draft. HL: Data curation, Funding acquisition, Methodology, Supervision, Writing – review & editing. HF: Conceptualization, Data curation, Investigation, Methodology, Writing – review & editing. XZ: Data curation, Methodology, Writing – review & editing. LWa: Conceptualization, Investigation, Resources, Writing – review & editing. LY: Project administration, Validation, Visualization, Writing – review & editing. LWu: Conceptualization, Data curation, Investigation, Writing – review & editing.
